# Associations between Polyfluoroalkyl Substances Exposure and Breast Cancer: A Meta-Analysis

**DOI:** 10.3390/toxics10060318

**Published:** 2022-06-11

**Authors:** Haihong Jiang, Huan Liu, Ge Liu, Jing Yu, Nana Liu, Yunqin Jin, Yongyi Bi, Hong Wang

**Affiliations:** School of Public Health, Wuhan University, Wuhan 430071, China; jiang-hh@whu.edu.cn (H.J.); lhlmlzw@126.com (H.L.); liuqiuge876@163.com (G.L.); yujing@whu.edu.cn (J.Y.); liunana@whu.edu.cn (N.L.); jinyq0513@163.com (Y.J.); yongyibi@aliyun.com (Y.B.)

**Keywords:** polyfluoroalkyl substance, breast cancer, pollutant, meta-analysis

## Abstract

Polyfluoroalkyl substances (PFASs) are persistent pollutants that may cause breast cancer. However, associations between exposure to PFASs and the risk of breast cancer are controversial. We retrieved studies on the association between PFASs—perfluorooctanoic acid (PFOA), perfluorononanoic acid (PFNA), perfluorohexane sulfonic acid (PFHxS), and perfluorooctane sulfonic acid (PFOS)—and breast cancer risk in women from PubMed, Embase, and the Web of Science. The pooled odds ratios (ORs) or relative risks (RRs) and their 95% confidence intervals (CIs) were extracted or calculated from provided data. Moreover, subgroup and metaregression analyses were performed to distinguish the potential sources of heterogeneity between studies. Lastly, eight original studies were included in the meta-analysis. PFOA and PFHxS were positively correlated with breast cancer risk, and the pooled ORs (and 95% CIs) were 1.32 (1.19 and 1.46) and 1.79 (1.51 and 2.11), respectively. PFNA was negatively correlated with breast cancer risk and the pooled OR (and 95% CIs) was 0.76 (0.6 and 0.96), and PFOS was shown to have no correlation with breast cancer risk and the pooled OR (and 95% CIs) was 1.01 (0.87 and 1.17). All results were merged in a random-effects model with significant heterogeneities (*I*^2^ > 90%, *p* < 0.001). The results demonstrated that PFASs might be potential risk factors for breast cancer, and the compounds in low exposure levels could have a more harmful impact on human health.

## 1. Introduction

Per- and polyfluorinated alkylated substances (PFASs) are a wide group of synthetic compounds that are water and oil repellent, and have been used in many industrial and consumer applications [1]. Direct exposure to these products can be quickly phased out by shifts in chemical production, but exposures driven by PFAS accumulation in the ocean, marine food chains, and the contamination of groundwater persist over long timescales [2]. PFASs are characterized by long half lives in biota and humans, and biomonitoring studies have suggested that two main PFASs, namely, PFOA and PFOS, are ubiquitously present in human blood [3]. Children exposed to PFASs might be associated with six categories of health outcomes: immunity/infection/asthma, cardio-metabolic, neurodevelopmental/attentional, thyroid, renal, and puberty onset [4]. Epidemiological studies have also revealed associations between exposure to specific PFASs and various health effects, including altered immune and thyroid function, liver disease, lipid and insulin dysregulation, kidney disease, adverse reproductive and developmental outcomes, and cancer [5].

PFASs are important environmental contaminants in drinking water, and have been linked to various adverse reproductive health outcomes in women [6]. A growing concern exists that exposure to chemical environmental contaminants, particularly EDCs, can lead to an increased incidence of breast cancer [7]. However, an evaluation of cancer risk with high exposure to various PFAS compounds in drinking water found no excess risk for breast cancer [8]. Similarly, some studies that focused on serum PFAS levels also found no association [9,10,11]. However, other studies indicated that PFASs are associated with breast cancer in a dose–response relationship [12,13]. The results of these epidemiological studies that investigated PFASs’ potential risk in breast cancer were inconsistent. Hence, the aim of the present meta-analysis based on previous studies was to elaborate the correlation between PFAS exposure and breast cancer risk.

## 2. Materials and Methods

### 2.1. Search Strategy

**Figure 1:**
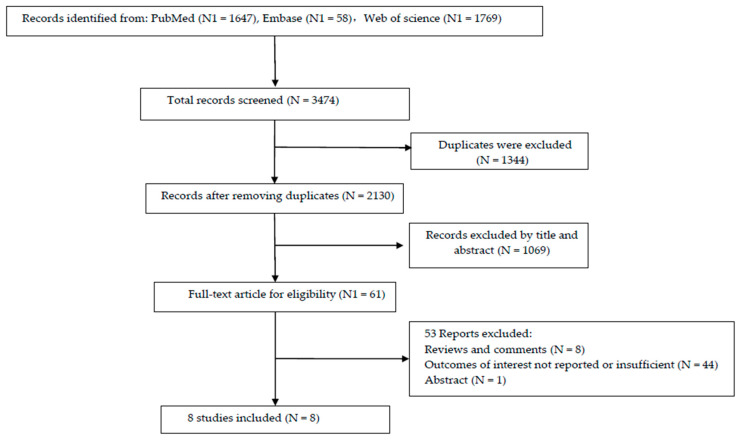
Flow chart of literature search and selection.

A systematic literature search was performed using PubMed, Embase, and Web of Science up to February 2022. The following relevant keywords were randomly combined to acquire the most comprehensive data: “Fluorocarbons” or “Perfluorocarbons” or “Fluorocarbon” or “Telomer Fluorocarbons” or “Fluorinated Telomer Alcohols” or “Fluorinated Telomer, Alcohols” or “Polyfluorinated Telomer Alcohols” or “Emulsion, Fluorocarbons” or “PFOA” or “PFNA” or “PFHxS” or “PFOS” and “cancers” or “tumors” or “neoplasms” or “malignancies”. A detailed search strategy is presented in Figure 1.

### 2.2. Inclusion and Exclusion Criteria

To identify eligible studies, specific criteria were used as follows: (1) restricted to English language articles and pathological breast cancer patients; (2) outcomes of interest were the association between PFASs and breast cancer; (3) OR, RR, or hazard ratio (HR) with 95% CIs of breast cancer were provided or could be calculated from the available data; (4) studies that measured exposures to PFOA, PFNA, PFUDA, PFHxS, and PFOS levels in blood. 

Additionally, the criteria for article exclusion were as follows: (1) studies focused on animal or cell experiments; (2) letters, reviews, editorials, case reports, or abstracts; (3) no data available to evaluate the correlation between PFAS exposure and breast cancer patients; (4) duplicates or samples used in previous publications.

### 2.3. Data Extraction and Quality Assessment

Data from all included studies were extracted by two reviewers (H.-H.J. and H.L.), and uncertain results were assessed by another investigator (H.W.). The extracted data included the following information: the first author, publication year, country, sample size, type of PFAS, study type, OR, RR, or HR, and 95% CIs. For significant heterogeneities among included studies, the random-effects model was used to merge results for meta-analysis.

Whether each included literature met the quality standards separately was assessed by two authors (H.-H.J. and H.L.). Any discrepancies were resolved through discussion and consensus. The quality of involved studies was evaluated with the Newcastle–Ottawa scale (NOS), which contains eight items and three dimensions [14]. The total score ranged from 0 to 9. Studies with over six points were considered high-quality, whereas those below four points were considered low-quality and were removed.

### 2.4. Statistical Analysis

In this meta-analysis, a combination of the pooled OR or RR and 95% CI was calculated to evaluate the relationship between PFASs and breast cancer. The study data were analyzed by Stata version 14-MP for Windows.

Subgroup analyses based on PFAS concentration levels, regions (Asian and Occident), and study type were performed to identify the potential sources of heterogeneity. Cochrane’s Q test and *I*^2^ statistics were applied to evaluate the heterogeneity of the pooled results [15]. *p* < 0.05 or *I*^2^ > 50% was recognized as statistically significant. If studies were proven to be homogenous, a fixed-effects model was utilized for further analysis. If not, the random-effects model was used. Finally, Begg’s and Egger’s tests were used to assess publication bias [16], and trim-and-fill analysis was used to examine the possible impact of publication bias [17]. Sensitivity analysis was also conducted by excluding one study at a time to determine the specific studies that significantly influenced the results.

## 3. Results

### 3.1. Literature Search Results

The entire process of the literature collection and screening is illustrated in Figure 1. Based on the search strategy, 3474 articles were identified through database probing. Then, 1344 duplicates were removed, and 2130 articles were excluded after reading the titles or abstracts. Finally, by carefully reviewing the full texts, eight original studies meeting the inclusion criteria were included in the meta-analysis [9,10,11,12,13,18,19,20]. The main features of the included studies in the meta-analysis are shown in Table 1.

### 3.2. Study Characteristics and Quality Assessment

The main characteristics of the eligible studies are summarized in Table 1. In the meta-analysis, the included populations came from six countries, including three Occident countries (Denmark, the US, and France) and three Asian countries (Japan, China, and the Philippines). PFASs mainly have four types, namely, PFOA, PFNA, PFHxS, and PFOS. The concentrations of these PFASs in serum were divided into three levels of exposure groups, including low-, medium- and high-exposure groups. Patient cases ranged from 75 to 905. They included two study types, with case–control studies reported on in six studies and one cross-sectional study.

Moreover, the detailed quality assessment of each study scored following the guidelines of NOS is shown in Table 2. All studies scoring over four points were incorporated in the meta-analysis.

### 3.3. Subgroup Analysis 

The subgroup analysis and metaregression were carried out to identify the potential sources of heterogeneity (Table 3). Results showed that the study type of included studies was the heterogeneity source of PFOA and PFHxS (*p* < 0.05). In addition, regions of these cases were the heterogeneity source of PFOA (*p* < 0.05). The concentration group might not have affected the reliability of the pooled results (*p* > 0.05).

### 3.4. Correlation between PFAS Exposure and Breast Cancer Risk

**Figure 2:**
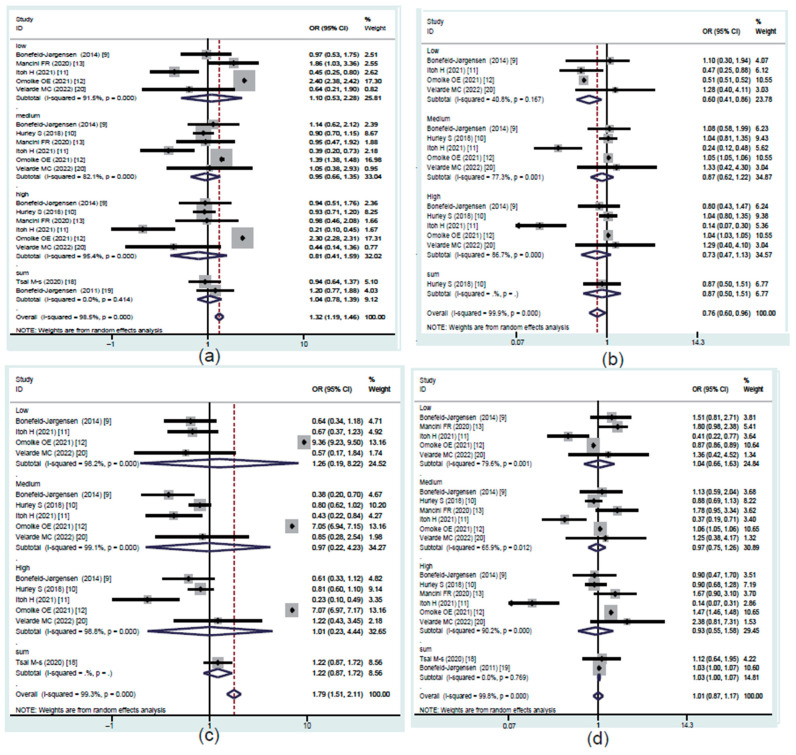
Forest plot of the studies that evaluated the ORs of PFASs. (**a**) PFOA; (**b**) PFNA; (**c**) PFHxS; (**d**) PFOS.

To evaluate the concentration-dependent difference in the association between PFASs and risk of breast cancer, studies were divided into three exposure groups on the basis of their quartile of the serum level of PFASs: Q2 (low), Q3 (medium), and Q4 (high). The pooled results are shown in Figure 2.

#### 3.4.1. PFOA Exposure and Prevalence Rate of Breast Cancer

**Figure 3:**
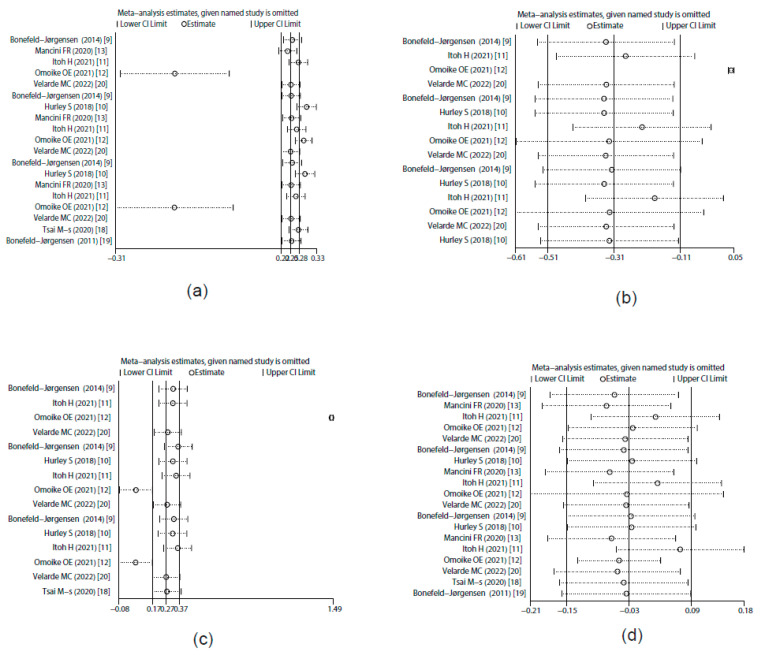
Sensitivity analysis of studies that evaluated the ORs of PFASs. (**a**) PFOA; (**b**) PFNA; (**c**) PFHxS; (**d**) PFOS.

**Figure 4:**
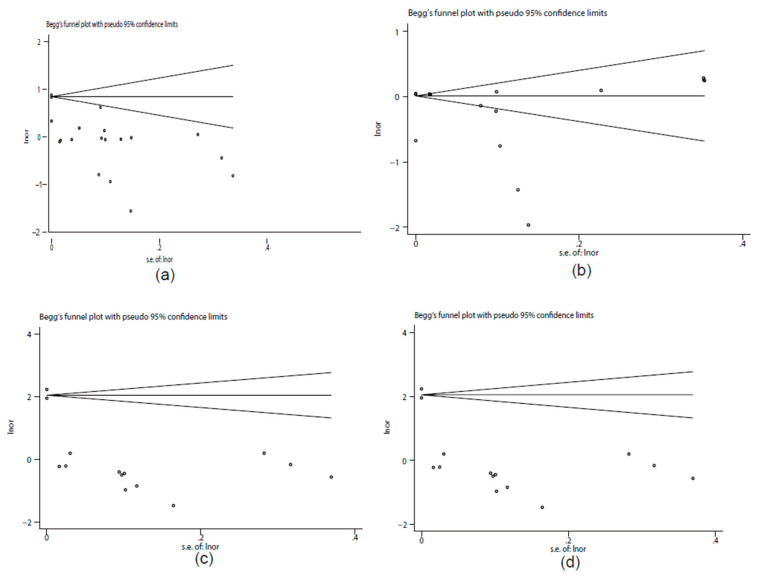
Begg’s funnel plot of publication bias. (**a**) PFOA; (**b**) PFNA; (**c**) PFHxS; (**d**) PFOS.

Eight studies provided data suitable for the meta-analysis of correlations between PFOA exposure and breast cancer risk (Figure 2a). The overall results showed that PFOA was positively correlated with breast cancer risk and the pooled OR was 1.32 (95% CI: 1.19, 1.46) with significant heterogeneity (*I*^2^ = 98.5%, *p* < 0.001). The subgroup analysis showed that different regions and study types of the included studies (*p* < 0.05) might have been important heterogeneity sources. The sensitivity analysis results revealed that the stability of the pooled OR might have been influenced by article [12], which was a cross-sectional study (Figure 3a). Begg’s funnel plot of PFOA was asymmetrical (Figure 4a). However, the *p* value of Egger’s test for PFOA was 0.659. This finding indicated that no publication bias existed in the included studies for PFOA. Through a trim-and-fill analysis, the pooled results were found to not be significantly affected for PFOA (OR = 1.283, 95% CI: 1.246, 1.321) compared with no trimming performed, indicating that the pooled OR was stable.

#### 3.4.2. PFNA Exposure and Prevalence Rate of Breast Cancer

Six studies provided data suitable for the meta-analysis of correlations between PFNA exposure and breast cancer risk (Figure 2b). The overall results showed that PFNA was negatively correlated with breast cancer risk and the pooled OR was 0.76 (95% CI: 0.6, 0.96) with significant heterogeneity (*I*^2^ = 99.9%, *p* < 0.001). The subgroup analysis failed to find the heterogeneity sources (*p* > 0.05). The sensitivity analysis results showed that the stability of the pooled OR might have also been influenced by article [12], which was a cross-sectional study (Figure 3b). Begg’s funnel plot of PFNA was asymmetrical (Figure 4b). However, the *p* value of Egger’s test for PFOA was 0.481. This finding indicated that no publication bias existed in the included studies for PFNA. Through a trim-and-fill analysis, the pooled results were different between merging models (the pooled OR was 1.012 (95% CI: 1.012, 1.012) in fixed-effects model, but 0.731 (95% CI: 0.55, 0.892) in random-effects model) and with no trimming performed, indicating that the pooled OR was unstable.

#### 3.4.3. PFHxS Exposure and Prevalence Rate of Breast Cancer

Six studies provided data suitable for the meta-analysis of correlations between PFHxS exposure and breast cancer risk (Figure 2c). The overall results showed that PFHxS was positively correlated with breast cancer risk and the pooled OR was 1.79 (95% CI: 1.51, 2.11) with significant heterogeneity (*I*^2^ = 99.3%, *p* < 0.001). The subgroup analysis showed that the different study types of included studies (*p* < 0.05) might have been primary heterogeneity sources. Additionally, the sensitivity analysis results showed that the stability of the pooled OR might have also been influenced by article [12], which was a cross-sectional study (Figure 3c). Begg’s funnel plot of PFHxS was asymmetrical (Figure 4c). However, the *p* value of Egger’s test for PFHxS was 0.904. This finding indicated that no publication bias existed in the included studies for PFHxS. Through a trim-and-fill analysis, the pooled results were found to not be significantly affected for PFHxS (OR = 1.31, 95% CI: 1.184, 1.442) with no trimming performed, indicating that the pooled OR was stable.

#### 3.4.4. PFOS Exposure and Prevalence Rate of Breast Cancer

Eight studies provided data suitable for the meta-analysis of correlations between PFOS exposure and breast cancer risk (Figure 2d). PFOS was not associated with breast cancer risk (OR = 1.01, 95% CI: 0.87, 1.17) with significant heterogeneity (*I*^2^ = 99.8%, *p* < 0.001). The subgroup analysis failed to find the heterogeneity sources (*p* > 0.05). The sensitivity analysis results showed that no outlier study was found (Figure 3d). Begg’s funnel plot of PFOS was asymmetrical (Figure 4d). However, the *p* value of Egger’s test for PFOS was 0.752. This finding indicated that no publication bias existed in the included studies for PFOS. Through a trim-and-fill analysis, the pooled results were found to be different between merging models (the pooled OR was 1.127 (95% CI: 1.127, 1.1272) in the fixed-effects model, but 1.01 (95% CI: 0.87, 1.17) in the random-effects model) with no trimming performed, indicating that the pooled OR was unstable.

## 4. Discussion 

In the meta-analysis, the results demonstrated that PFOA and PFHxS were positively correlated with breast cancer risk (OR = 1.32, 95% CI: 1.19, 1.46; and OR = 1.79, 95% CI: 1.51, 2.11, respectively), whereas the PFNA was negatively correlated with breast cancer risk (OR = 0.76, 95% CI: 0.6, 0.96) and PFOS was not associated with breast cancer risk (OR = 1.01, 95% CI: 0.87, 1.17). 

In the included studies, researchers found some common risk features of patient cases, such as a family history of breast cancer, smoking, BMI, full-term pregnancy, etc. Although there was significant heterogeneity, the pooled results of PFOA and PFHxS were stable and reliable. The pooled results of PFNA and PFOS were different between merging models, indicating that the pooled ORs were unstable, which might have been influenced by the quantities and qualities of the included studies. 

The causes of breast cancer are constantly being updated by researchers. Some established breast cancer risk factors include early age at giving first birth, genetic inheritance [21,22], an unhealthy lifestyle [23], and hazardous environmental exposure [24] that can explain a significant part of breast cancer incidence. Among the proposed risk factors, environmental chemical pollution may increasingly affect the signaling pathways involved in the emergence and progression of metastatic tumor cells. From the summarization by Meriem Koual [25], environmental chemicals play a great role in breast cancer progression, metastasis formation, and resistance to chemotherapy. PFASs have diverse toxicities, such as a potential carcinogenic nature [26], developmental toxicity [27], and endocrine-disrupting activities [28]. PFOA and PFOS have been listed as persistent organic pollutants (POPs) in the Stockholm Convention to restrict or eliminate their production and use to protect human health and the environment.

Some in vitro studies demonstrated that the exposure of human breast epithelial cells to PFOA and PFOS induced an increase in cell proliferation, cell migration, and invasion potential by differentially affecting proteins such as cell-cycle regulators, β-integrin, E-cadherin, and occlusion, as well as global DNA methylation and histone modifications [29]. PFOA also promotes human breast epithelial cell (MCF-10A) proliferation by accelerating the G0/G1to S phase transition of the cell cycle [30], and PFOS also has the same pathogenic mechanism in low exposure levels [31]. Exposure to PFOA can result in DNA demethylation accompanied by altered expression patterns of DNA methyltransferase and an increased susceptibility to cancer [32]. Estrogen receptor (ER) upregulation has been associated with tumor progression and is the most used clinical biomarker in breast cancer. In ER-positive cell lines, 17 beta-estradiol (E2) results in a complex formation between ERα, CREB-binding protein, and BRCA1 to reproduce the effects on DNA damage, repair, and survival [33]. The activation of the MAPK pathway might be a molecular target of PFOS and PFOA to promote E2-induced T47D cell line growth [34]. Short-chain (100 μM) PFHxS affects important regulatory cell-cycle proteins (cyclin D1, CDK6, p27, p53, and ERK) and induces cell proliferation in some part through the activation of the constitutive androstane receptor and the peroxisome proliferator-activated receptor alpha [35]. PFNA can impact normal acini maturation at low concentration, causing the loss of the organization of cell clusters and the absence of hollow lumen [36]. Overall, PFASs can interfere with cellular events regarding the normal development of glandular breast tissue.

The epidemiological studies on the association between PFAS exposure and breast cancer risk are usually diverse. A study from China found that PFHxS and PFOS positively correlated with ER+ breast cancer risk in younger women [18]. Likewise, the EN3 study from France indicated that PFOS concentration levels were positively associated with ER+ breast cancer risk, whereas PFAS concentrations were not associated with breast cancer risk overall [13]. Meanwhile, a case–control study that evaluated the association between the serum levels of PFAS in pregnant women in Denmark during a follow-up period of 10–15 years demonstrated that weak positive and insignificant negative associations were found between breast cancer risk, PFOS, and PFHxS [9]. The California Teachers study failed to provide evidence that PFAS levels measured after diagnosis are related to breast cancer risk [10]. 

PFASs might increase the risk of disease by disrupting hormone-mediated processes. However, the impact of the mixtures of environmental chemicals along the carcinogenic process can be triggered or boosted by individual chemicals through different mechanisms [37]. 

The studies included in this meta-analysis exhibited differences, which might have caused heterogeneity. In addition, most of the included studies were case–control studies, which had some limitations. Case–control studies are always retrospective, which could introduce bias during the recollection of samples for exposure estimations [38], and they could just prove a correlation but not reveal any causation [39]. 

A cross-sectional study was also incorporated in this meta-analysis, and the subgroup-analysis results showed that the study might have been an important heterogeneity source. The outcome of breast cancer (yes/no) was determined without clinical confirmation evidence measuring recruited patients, which was significantly different from other included studies [12]. Nevertheless, this article met the inclusion and quality criteria, so we did not exclude it. Some other features exist that caused the heterogeneity, but because of the limited information provided by the original studies, we could not conduct an in-depth analysis to demonstrate the specific correlations among them, illuminating some parts of heterogeneity in the study.

## 5. Conclusions

Our meta-analysis provided evidence suggesting that PFASs, especially PFOA and PFHxS, might be potential risk factors of breast cancer. Although the results show that PFOS is not associated with breast cancer risk and PFNA is negatively associated with the risk, in the context of some in vitro studies, PFOS and PFNA are potential risk factors for breast cancer. In addition, PFOS and PFOA are mostly detected in aquatic environments, and our results showed that compounds in low-exposure levels would have a more harmful impact on human health. Therefore, more well-designed large studies, especially some prospective studies, are warranted to further explore the association between PFAS exposure (single and combined) and breast cancer risk to uncover the underlying biological mechanism for preventing breast cancer and other PFAS-related diseases.

## Figures and Tables

**Table 1:** Characteristics of studies entered in the meta-analysis.

First Author	Country	Patient	OR/RR (95% CIs) Study				Study
Year		Cases	PFOA	PFNA	PFHxS	PFOS	Type
Bonefeld-Jørgensen (2014) [9]	Denmark	250	0.97 (0.53, 1.75) ^a^ 1.14 (0.62, 2.12) ^b^ 0.94 (0.51, 1.76) ^c^	1.10 (0.30, 1.94) ^a^ 1.08 (0.58, 1.99) ^b^ 0.80 (0.43, 1.47) ^c^	0.64 (0.34, 1.18) ^a^ 0.38 (0.20, 0.70) ^b^ 0.61 (0.33, 1.12) ^c^	1.51 (0.81, 2.71) ^a^ 1.13 (0.59, 2.04) ^b^ 0.90 (0.47, 1.70) ^c^	#
Hurley S (2018) [10]	The US	902	NR 0.901 (0.705, 1.152) ^b^ 0.925 (0.715, 1.197) ^c^	NR 1.043 (0.808, 1.345) ^b^ 1.037 (0.798, 1.348) ^c^	NR 0.798 (0.621, 1.025) ^b^ 0.801 (0.619, 1.035) ^c^	NR 0.883 (0.691–1.129) ^b^ 0.898 (0.695, 1.161) ^c^	#
Mancini FR (2020) [13]	France	194	1.86 (1.03, 3.36) ^a^ 0.95 (0.47, 1.92) ^b^ 0.98 (0.46, 2.08) ^c^	NR NR NR	NR NR NR	1.80 (0.98, 3.28) ^a^ 1.78 (0.95, 3.34) ^b^ 1.67 (0.90, 3.10) ^c^	#
Itoh H (2021) [11]	Japan	401	0.45 (0.25, 0.80) ^a^ 0.39 (0.20, 0.73) ^b^ 0.21 (0.10, 0.45) ^c^	0.47 (0.25, 0.88) ^a^ 0.24 (0.12, 0.48) ^b^ 0.14 (0.07, 0.30) ^c^	0.67 (0.37, 1.23) ^a^ 0.43 (0.22, 0.84) ^b^ 0.23 (0.10, 0.49) ^c^	0.41 (0.22, 0.77) ^a^ 0.37 (0.19, 0.71) ^b^ 0.14 (0.07, 0.31) ^c^	#
Omoike OE (2021) [12]	The US	11631	2.40 (2.38, 2.42) ^a^ 1.39 (1.38, 1.40) ^b^ 2.30 (2.28, 2.31) ^c^	0.51 (0.51, 0.52) ^a^ 1.05 (1.05, 1.06) ^b^ 1.04 (1.03, 1.05) ^c^	9.36 (9.23, 9.50) ^a^ 7.05 (6.94, 7.15) ^b^ 7.07 (6.97, 7.17) ^c^	0.87 (0.86, 0.89) ^a^ 1.06 (1.05, 1.06) ^b^ 1.47 (1.46, 1.48) ^c^	*
Tsai M-s (2020) [18]	China	119	0.94 (0.64, 1.37)	0.87 (0.50, 1.51)	1.22 (0.87, 1.72)	1.12 (0.64, 1.95)	#
Bonefeld-Jørgensen (2011) [19]	Denmark	31	1.20 (0.77, 1.88)	NR	NR	1.03 (1.001, 1.07)	#
Velarde MC (2022) [20]	The Philippines	75	0.64 (0.21, 1.90) ^a^ 1.05 (0.38, 2.93) ^b^ 0.44 (0.14, 1.36) ^c^	1.28 (0.40, 4.11) ^a^ 1.33 (0.42, 4.30) ^b^ 1.29 (0.40, 4.10) ^c^	0.57 (0.17, 1.84) ^a^ 0.85 (0.28, 2.54) ^b^ 1.22 (0.43, 3.45) ^c^	1.36 (0.42, 4.52) ^a^ 1.25 (0.38, 4.17) ^b^ 2.38 (0.81, 7.31) ^c^	#

PFOA: perfluorooctanoic acid; PFNA: perfluorononanoic acid; PFHxS: perfluorohexane sulfonate; PFOS: perfluorooctane sulfonate; OR: odds ratio RR: relative risk; ^a^: the result for the low-level exposure group; ^b^: the result for the medium-level exposure group; ^c^: the result for the high-level exposure group; NR: not researched; #: case–control study; *: cross-sectional study.

**Table 2:** Newcastle–Ottawa quality assessment scale.

First Author	Quality Indicators from Newcastle–Ottawa Scale								Scores
(Year)	1	2	3	4	5	6	7	8	
Bonefeld-Jørgensen (2014) [9]	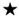	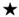	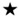	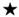	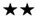	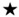	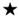	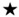	9
Hurley S (2018) [10]	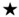	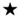	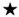	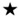	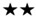	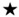	-	-	7
Mancini FR (2020) [13]	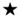	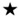	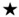	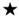	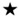	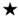	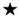	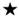	8
Itoh H (2021) [11]	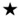	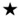	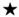	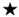	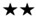	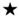	-	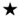	8
Omoike OE (2021) [12]	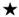	-	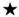	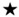	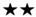	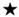	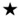	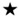	8
Tsai M-s(2020) [18]	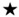	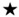	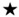	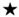	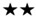	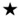	-	-	7
Bonefeld-Jørgensen(2011) [19]	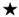	-	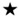	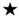	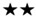	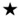	-	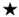	7
Velarde MC (2022) [20]	-	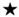	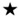	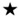	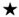	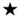	-	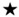	6

1. Representativeness of the exposed cohort (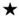); 2. selection of the nonexposed cohort (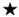); 3. ascertainment of exposure (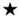); 4. outcome of interest was not present at start of study (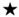); 5. control for important factor (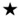) or/and additional factor (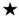); 6. assessment of outcome (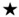); 7. follow-up long enough for outcomes to occur (≥5 years) (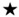); 8. adequacy of follow-up of cohorts (lost follow-up ≤ 25%) (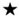).

**Table 3:** Subgroup analysis and metaregression of PFASs.

Subgroup	PFOA				PFNA			
	Studies	Pooled ORs	Heterogeneity	*p* of	Studies	Pooled ORs	Heterogeneity	*p* of
	(N)	(95% CI)	(*I*^2^, *p*)	Metareg	(N)	(95% CI)	(*I*^2^, *p*)	Metareg
Concentration Group								
Sum	2	1.04 (0.78, 1.39)	0.00, 0.414		1	0.87 (0.74, 1.02)	0.00	
Low	5	1.10 (0.53, 2.28)	91.5%, <0.001	0.909	4	0.60 (0.41, 0.86)	40.8%, 0.167	0.777
Medium	6	0.95 (0.66, 1.35)	82.4%, <0.001	0.790	5	0.87 (0.62, 1.22)	77.3%, <0.001	0.945
High	6	0.81 (0.41, 1.59)	95.4%, <0.001	0.661	5	0.73 (0.47, 1.13)	87.6%, <0.001	0.784
Regions								
Asian	3	0.52 (0.33, 0.82)	64.5%, <0.001		2	0.56 (0.29, 1.07)	78.4%, <0.001	
Occident	5	1.53 (1.38, 1.71)	98.9%, <0.001	0.001	3	0.91 (0.68, 1,23)	100%, <0.001	0.146
Study type								
Cross-sectional	1	1.98 (1.74, 2.24)	99.8%, <0.001		1	0.82 (0.54, 1.26)	100%, <0.001	
Case–control	7	0.83 (0.67, 1.02)	58.6%, 0.002	0.01	7	0.73 (0.51, 1.03)	76.6%, <0.001	0.784
	PFHxS				PFOS			
Concentration Group								
Sum	1	1.22 (1.15, 1.29)	0.00		2	1.03 (1.00, 1.07)	7.0%, 0.300	
Low	4	1.26 (0.19, 8.22)	98.2%, <0.001	0.964	5	1.04 (0.66, 1.63)	79.6%, <0.001	0.796
Medium	5	0.97 (0.22, 4.23)	99.1%, <0.001	0.878	6	0.97 (0.75, 1.26)	65.9%, <0.001	0.899
High	5	1.01 (0.23, 4.44)	98.8%, <0.001	0.904	6	0.93 (0.55, 1.58)	90.2%, <0.001	0.792
Regions								
Asian	2	0.65 (0.40, 1.08)	69.9%, <0.001		2	0.67 (0.33, 1.34)	81.4%, <0.001	
Occident	3	2.66 (2.22, 3.20)	99.6%, <0.001	0.122	4	1.14(0.97, 1.34)	99.8%, <0.001	0.058
Study type								
Cross-sectional	1	7.76 (6.45, 9.33)	99.8%, <0.001		1	1.11 (0.85, 1.44)	100%, <0.001	
Case–control	7	0.67 (0.52, 0.86)	57.5%, 0.007	0.004	7	0.96 (0.77, 1.20)	76.2%, <0.001	0.737

## Data Availability

The authors confirm that the data supporting the findings of this study are available within the article.
